# A sustainability scoreboard for crop provision in Europe

**DOI:** 10.1016/j.ecoser.2020.101194

**Published:** 2020-12

**Authors:** Silvia Cerilli, Alessandra La Notte, Domenico Pisani, Sara Vallecillo, Francesco Nicola Tubiello

**Affiliations:** a Food and Agriculture Organization of the United Nations (FAO), Via delle Terme di Caracalla, 00100 Rome, Italy; b European Commission - Joint Research Centre, Via Enrico Fermi 2749, 21027 Ispra, VA, Italy; c IUAV - University of Venice, Via Santa Croce 191, 30135 Venezia, VE, Italy

**Keywords:** Crop provisioning, Environmental accounts, Ecosystem services, Composite indicators, Sustainability scoreboards

## Abstract

The System of Environmental-Economic Accounting for Agriculture, Forestry and Fisheries (SEEA AFF) offers the possibility to assess and report detailed accounts for primary industries while establishing important linkages with relevant ecosystem services, in line with the SEEA Experimental Ecosystem Accounting (SEEA EEA). In this paper, crop products and crop provision as ecosystem service are coherently merged to build a sustainability scoreboard for selected crops in European countries. The sustainability scoreboard uses the SEEA AFF accounts for crop products with data inputs from FAOSTAT and the Integrated system of Natural Capital Accounts (INCA) of the Joint Research Centre (JRC) for ecosystem services. The combined FAO-JRC accounting table described in this paper provides a common ground and measurement tool towards a sustainability scoreboard, useful to analyse how relevant economic, social and environmental components behave by country. This newly derived sustainability scoreboard presents significant differences with respect to analyses based on standard agricultural statistics. Lack of sufficiently accurate data remains the major limitation to current fuller implementation of the sustainability scoreboard. However reasonable assumption can be made that ongoing international data collection processes (including FAO questionnaires) integrated with Geographic Information System (GIS) analysis will supply in the near future additional relevant and applicable information.

## Highlights

• This paper links international statistical environmental-economic standards for natural resources and ecosystem service accounts.
• In accounting for crop provision, an emergy approach is applied to identify and disentangle natural inputs from anthropogenic factors and measure ecosystem contribution to this service.
• Moreover, a sustainability scoreboard is derived to analyse how relevant economic, social and environmental components behave by country and by crop and to align this analytical effort with the international call for the Sustainable development Goals (SDG), including SDG 12, Ensure sustainable consumption and production patterns, and SDG 15, Life on land, which promotes the sustainable use of terrestrial ecosystems as essential natural resources for economy and society.

## 1 Introduction

Farming can take place with a variety of management practices: from intensive (high human input) to organic and extensive (low human input) agriculture.^1^ Data on total agricultural production alone provide critical information on some of the traditional pillars of sustainability (UN, 1987, UNEP, 2017, UNEP, UNSD, CBD, SEEA). As already pointed out in (Sukhdev et al., 2016, La Notte et al., 2017), evaluating agriculture and food systems requires understanding of a vast and interacting complex of ecosystems and social and economic actors. The Economics of Ecosystems and Biodiversity (TEEB) initiative for Agriculture & Food published important studies on the importance to evaluate the nexus between the agri-food sector, ecosystem services and externalities on human health (“The Economics of Ecosystems and Biodiversity TEEB,” 2018). This paper proposes a sustainability scoreboard for crop provision based on an integrated framework, in line with current environmental economic accounting developments by the international community.

The United Nations Statistical Commission adopted, at its 43rd Session in 2012, the System of Environmental-Economic Accounting Central Framework (SEEA CF) as the first international statistical standard for environmental-economic accounting (United Nations, 2014). The SEEA Agriculture Forestry and Fisheries (SEEA AFF) extends the SEEA CF accounting framework by detailing the structure of the accounting records concerning all economic activities covering agriculture, forestry and fisheries (classified as International Standard Industrial Classification, ISIC, A) and their relationship with the environment (FAO and UNSD, 2020).

The SEEA AFF defines core national accounting tables for the measurement and reporting of information on physical and monetary assets and flows covering natural resource use, production, trade and consumption of food and other agricultural products. It thus represents a robust statistical structure useful for the development of agri-environmental indicators, including the SDGs, which are internationally comparable. The SEEA AFF includes a specific accounting table for crop production, the Physical flow account for crops (ref. Table 3.1, in FAO and UNSD, 2020). This account is used in this analysis as starting point to measure crops as System of National Accounts (SNA) products. In fact, the physical flow account for crops records the supply and use of food and non-food crop products in physical terms, usually tonnes. For each product – rice, for example – the table records: i) total supply of the raw product from the agriculture industry, and from the rest of the world; ii) total use of the raw product, for example intermediate consumption to the manufacturing sector or to export; iii) total supply of the processed product; and iv) total use of the processed product, including household consumption. The recording of supply- and -use flows of crops in both raw and processed forms enables a link with household consumption of food products, and hence the information can support assessment of food security and nutrition. (Further details on the SEEA AFF accounting tables for crop products may be found at: SEEA AFF, chapter 3.2, paragraphs 3.12–3.55).

Ecosystem contribution to the growth of biomass is not included within the scope of the SNA nor the SEEA CF. In fact, the crop products enter the economy and contribute to the gross domestic product (GDP), being anything else part of the “environment” in the SEEA CF and in the SEEA AFF. The SEEA EEA complements the SEEA CF conceptual framework, by attempting to record the ecosystems contributions to standard measures of economic activity (such as GDP, and national income), while also quantifying the concomitant provision of a range of other benefits to human well-being that are commonly unpriced and not considered in national accounting. In the SEEA EEA accounting framework are measured SNA and not SNA benefits. While SNA benefits are already fully measured in the SEEA CF accounting system, an important rationale for measuring ecosystem services is the understanding that there are many other, non-SNA, benefits that economic units, and society more generally, receive from ecosystem assets, especially in the agricultural sector. More specifically, for ecosystem accounting, the production boundary is expanded relative to the SNA, reflecting that the supply of goods and services by ecosystems is considered additional production (Eigenraam and Obst, 2018; La Notte et al., 2019). In this perspective, this paper explores and analyses ecosystem cropland contribution to crop products through the crop provisioning service. It supports the analysis perspective as highlighted in the SEEA EEA Technical Recommendation (United Nations, 2017): “An important part of the rationale for measuring ecosystem services is that much economic production utilizes inputs directly taken from ecosystems but these inputs (and any associated costs of capital) are not recorded in the standard accounting framework.^2^” Crop provisioning as ecosystem service is in fact the accounting link between ecosystem assets and economic activity. The European Commission applies and contribute to the SEEA EEA development in its Knowledge Innovation Project on an “Integrated system of Natural Capital and ecosystem services Accounting’’ (INCA) initiative, with the objective of building biophysical and monetary accounts at European Union (EU) level, to respond to EU policies. As part of the INCA project, the Joint Research Centre (JRC), among other European Commission services has assessed crop and timber provision by applying a procedure that aims at disentangling the contribution of ecosystem in generating biomass from human input co-joint action. In the case of crop provision, an *emergy*^3^ based approach is applied to separate natural input (such as sun, rain, soil) from human input (as fertilizer, irrigation, machinery) to disentangle ecosystem contribution from total crop yield. In fact, a high crop production can be the result of intensive agricultural practices, where ecosystem contribution (in relative terms) is very low. On the other hand, extensive agricultural practices likely record high ecosystem contribution, where ecosystems have a more active role contributing to the yield production in relative terms.

This approach is in line with the definition of ecosystem service, although it is usually not considered when reporting the flow of crop provision (Marta-Pedroso et al., 2018, Power, 2010, Schipanski et al., 2014). The JRC supply and use tables track the flow of services from the ecosystem to the primary sector and to other commonly unpriced human activities; in this article we only consider the crop provision service to the agricultural sector (Vallecillo et al., 2019; Pérez-Soba et al., 2019). In performing this methodological advancement from the only SEEA CF approach, combining JRC Ecosystem Services (ES) accounts with the FAO AFF ensures consistency with the underlying SNA and SEEA accounting framework, considering that the latter is already used at EC level for country reporting of specific SEEA accounts.

Merging the FAO Physical Supply and Use Table for Crops and the JRC Terrestrial Provisioning Service Table, a new FAO-JRC accounting table is derived, which is compliant via SEEA AFF to the SEEA CF and the SNA and via JRC Terrestrial Provisioning Service to the SEEA EEA. The crop provisioning accounting table records under the field ISIC economic units the output of agricultural (ISIC A 01) and manufacturing industries (ISIC C 10). Therefore, the accounting system allows keeping track of the role of the ecosystem in the whole agricultural production chain, from the raw commodity to the processed product, which constitutes an SNA benefit for final consumers. By reproducing the SEEA CF and the SNA accounting structures, flows from the rest of the world are also recorded.

It has to be noticed that the accounting structure allows to record values by crop or by crop types.^4^ Crops and their aggregates are compliant with the Central Product Classification (CPC),^5^ which constitutes a complete product classification covering all goods and services. It serves as an international standard for assembling and tabulating all kinds of data requiring product detail, including statistics on industrial production, domestic and foreign commodity trade, international trade in services, balance of payments, consumption and price statistics and other data used within the national accounts. It provides a framework for international comparison and promotes harmonization of various types of statistics related to goods and services. Moreover, crop as presented in the FAO-JRC accounting table are also compliant with the FAOSTAT Commodity List (FCL) which contains internationally recognized definitions, concepts and classifications of commodities recorded in FAOSTAT and the INCA classification system as shown in Annex I. Therefore the resulting FAO-JRC combined accounting table allows to record not only raw crop production but also processed crops and crop trading. It therefore facilitates the analysis of ecosystem contribution to the whole crop production supply system.

In merging AFF and INCA, the paper aims to quantify the ecosystem contribution to agricultural sector in terms of intermediate and final consumption and therefore contributes to the international research agenda, as the Glen Cove one. From the 18th to 20th of June 2018, national and environmental accountants, researchers and experts in the areas of ecosystem accounting, ecosystem modelling, spatial analysis of ecosystem services, scientists, and environmental and ecological economics, earth observation specialists met in Glen Cove, New York, USA for the 2018 Forum of Experts in SEEA Experimental Ecosystem Accounting. The forum dealt four research areas: (1) Spatial areas (2) Ecosystem condition (3) Ecosystem services (4) Accounting treatments and valuation. I particular research area (3) focused on measuring ecosystem service flows in physical terms to enable a broad mapping of the role of ecosystem assets and the relevant beneficiaries; and to facilitate the valuation of ecosystem services. This paper, by analysing a specific ecosystem service (namely the crop provisioning one) attempts to contribute to the area 3, Ecosystem Services. Through a sustainability scoreboard it is in fact possible to merge analytical perspectives (such the market, the ecological and the social ones) into a consistent and coherent framework and thus analyse data concerning the agricultural and food systems into a broader context. Despite some data limitations to provide comprehensive measures of these scores,^6^ this indicator can be a robust and useful tool not only for the relevant information on economic, social and environmental aspects that can be derived, but also in the comparability across countries that can be reached thought its compliancy with main international standard for environmental economic accounting (as the SEEA). Finally, in describing the crop provisioning service and the derived sustainability scoreboard, this paper also suggests indicators that may be derived from this accounting framework and how it may support the measurement of few SDGs such as zero hunger (SDG 2), economic growth, responsible production and consumption (SDG 12), and life on earth (SDG 15).

## 2 Methods

The System of Environmental-Economic Accounting for Agriculture Forestry and Fisheries (SEEA AFF) expands the international statistical standard for environmental-economic accounting, the SEEA-CF, to the International Standard Industrial Classification of All Economic Activities (ISIC) agricultural activities (namely, ISIC A 01, Crop and Livestock; 02 Forestry; 03 Fishing). Developed by FAO in collaboration with UNSD and other international and national partners, the SEEA AFF is an internationally agreed methodological document (UNCEEA/11/1, June 2016) in support of the SEEA CF and as and such it is compliant with the SNA (EC et al, 2009). As added value, its tables can be easily compiled with FAOSTAT data as a default approach, when relevant national data are missing.

Ecosystem services accounts developed by JRC under the INCA umbrella are consistent with the SEEA 2012 Experimental Ecosystem Accounting (SEEA EEA, UN et al. 2014b) and its Technical Recommendations (United Nations, 2017). The joint FAO-JRC analysis described in this paper allows the development of specific crop provisioning service accounts, which represent an extension of the SEEA AFF crop production accounting table (in line with the SNA and the SEEA CF) and the corresponding JRC terrestrial provisioning service accounts, which are compliant with the SEEA EEA.

A combined accounting table merging AFF from FAO and INCA from JRC is therefore consistent with the SEEA EEA, the SEEA CF and the SNA. After initial screening, a simplified procedure (i.e. by using FAOSTAT and JRC-Eurostat database) is formulated for crop provisioning service and its biomass production recording and analysis. More specifically, this paper assesses the contribution of the ecosystem type cropland to the SNA benefits received by agricultural products consumers. Crop provision accounts from INCA estimate the ecosystem contribution as natural energy sources (including solar radiation, water and soil mineral resources), separated from anthropogenic resources, such as fertilizers, irrigation and machinery (for more details ref. chapter 3 in Vallecillo et al. (2019).

**Fig. 1:**
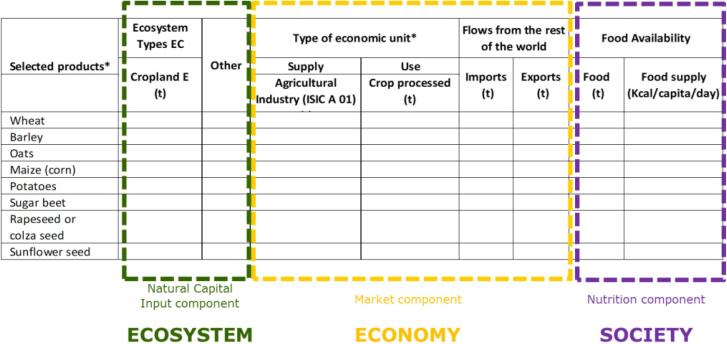
Structure of the FAO-JRC combined accounting table.

Fig. 1 shows the three analytical pillars and related representative variables of the FAO-JRC combined accounting table, namely: (I) ecosystem (actual flow of crop provision as ecosystem contribution), (II) economy (agricultural production and manufacturing, imports and exports) (iii) society (food availability).^7^

These clearly define a sustainability concept in line with main UN international guidelines, as the 2030 Development Agenda and its 17 Sustainable Development Goals (SDGs).^8^ This plan for action recognises that eradicating poverty in all its forms and dimensions, is the greatest global challenge and indispensable requirement for sustainable development. At the same time, it offers a clear definition of sustainable development, which consists of three dimensions: economic, social and environmental. These dimensions need to be achieved in a “balanced and integrated manner”.^9^ Moreover, the agenda for sustainable development is structured in specific targets (169) and indicators (131) that constitute a clear framework for measuring these goals, and to which our scoreboard attempt to contribute (and in particular to SDG 8, 12 and 15, as shown in Section 3, discussion).

In terms of accounting structure, as shown in Fig. 1 the ecological and social components are available respectively from INCA ES accounts and FAO Food Balance Sheets. For the market component, we attempt to build a composite indicator (described in Section 2.1). To this end, we selected those components where increasing amounts are preferable to decreasing amounts, i.e. a positive trend is desirable for all of them. Higher production of the crop is a resource for the country. This resource can further increase richness by transforming the raw product into a product with higher value added, or by trading it.

The primary source of data is FAOSTAT. Crops are selected based on the availability of INCA accounts on ecosystem contribution. Further selection applied to establish a correspondence between INCA-ESTAT classification and FAO classification. Annex I reports in details crop mapping among different systems. Fig. 1 describes main cereals (wheat, barley, and oats), oil bearing crop (rapeseed, sunflower seed), roots and tubers (potatoes) and sugar crop (sugar beet) used in this exercise. They represent the main commodity in terms of national crop agricultural output.

The correspondence between raw crops and processed crops is adopted from FAOSTAT and reported in Annex II. The use of processed crop is important to understand how much of the raw crop is used as intermediate production to generate goods with higher value added.

ESTAT datasets are used in parallel with a double purpose: (i) to validate FAO data and check whether major differences occur, (ii) to integrate whether appropriate missing data.

FAO-JRC combined accounting tables are reported for each crop as Supplementary Material.^10^ In the following section the composite indicator for the market component is explained. We have to build a composite indicator because we need to combine different information into one indicator. Then the scoreboard is described. We decide to use a scoreboard because the three pillars of sustainability could show some trade-off; the fact that they could not follow the same path does not prevent us to build a composite indicator.

### 2.1 Building the composite indicator for the market assessment

The rules followed here to build the composite indicator are: (i) choose sub-indicators with similar trends in terms of direction (i.e. higher implies an improvement for all the variables chosen), (ii) use a common unit meaningful in terms of market figures (i.e. everything is reported in monetary terms), (iii) avoid the size effect (i.e. big countries have higher values) and thus always use relative values. As already mentioned, the sub-indicators are: raw crops, processed crops, export and import.

The first step was to analyse the dataset in order to assure that countries are taken into account for the crops they produce:

• by confronting the two datasets it is possible to validate the fact that missing data means that a specific country does not produce a specific crop, and it is not a problem of data gaps;
• by confronting individual numbers, it is possible to validate similar order of magnitude or highlight where there are inconsistencies;
• where data gaps are recorded, the European Statistics database (ESTAT) dataset can be used to fill missing data in FAOSTAT, as appropriate.

The only crop with complete data for all the countries was barley. For wheat, the countries not included in the table were Finland, Luxembourg and Slovenia. For maize, the countries not included in the table were Estonia, Ireland, Latvia and Finland; estimates were undertaken for UK using the ESTAT data. Sweden, Belgium, Denmark and Luxembourg were not considered because data on processed crops were missing (we refer from here onwards to EU-24 as the study area). For rapeseed the only country not considered was Portugal. For sugar beet, Bulgaria, Estonia, Ireland, Luxembourg, Latvia and Slovenia are not included in the table. We need to report that Finland records zero value on export, and Denmark, Poland and Sweden report zero value on import. For sunflower, Belgium, Denmark, Estonia, Finland, Ireland, Lithuania, Luxembourg, Latvia, Netherlands, Sweden, Slovenia and UK are not included in the table. For oats and potatoes, the market indicator is calculated without considering processed crops because data are completely missed.

Data in physical terms are multiplied by a unit price value. In FAOSTAT, “Output price at farm gate ($/t)” is available for each crop in each country. However, when this estimate is not available, the EU average is used.^11^ Once physical production amounts (in metric tonnes, t) are multiplied by unit price, the outcome is divided by GDP (US$ thousands^12^). The use of GDP as denominator has a double impact: it resolves the size effect and it weights the importance of the agricultural sector for the country overall economy.

The variables that are used to build the composite indicator are

• raw crop as output (US$)/GDP (US$ thousand): relative value of raw crops in monetary terms;
• processed crop (US$)/GDP (US$ thousand): relative value of processed crops in monetary terms. As already mentioned for oats and potatoes this sub-indicator is not calculated because of data gaps;
• export (US$) – import (US$)/GDP (US$ thousand): relative value of traded crops in monetary terms.

Following the methodology suggested by COIN (ref. https://composite-indicators.jrc.ec.europa.eu/?q=about-us) and described in OECD (2005), the following steps are applied:

• Data treatment, by imputing missing data (through the integration with the ESTAT dataset and the use of averages) and treating outliers;
• Assessment of the statistical and conceptual coherence, by performing correlation analysis;
• Normalization, by applying the following equation:

(1) $$\mathrm{Normalization}=\frac{\left(x-\min x\right)}{\left(\max x)-\min x\right)}$$

• Aggregation, once normalized the variables are aggregated by using the equal weight of 0.333^13^ for all crops except oats and potatoes (for which a weight of 0.5 is used because we could not include the element “processed crops”):

(2) $$\mathrm{Mkt}=\prod \pi^{w}$$

where π=variables, and w = weight.

• Final ranking.

### 2.2 Building the sustainability scoreboard

Once the composite indicator is built for the market component, we have all the elements we need to create a sustainability scoreboard. Before proceeding with the comparison, we have to normalize the ecosystem contribution element and the food supply element by using the same formula used for the market component (ref, Eq. (1)) to be consistent.

Please be aware that ecosystem contribution in this case only refers to natural input provided by ecosystem to agricultural production (such as sun, rain, soil), separated by human input (such as fertilizers and pesticide, irrigation, use of machinery). Of course, ecosystems provide also multiples other regulating and recreational services to the environment and human beings: e.g. water purification cleans the excess of nitrogen used in fertilizers and flood control protects cropland from the risk of flooding. However, for this exercise we limit our assessment to the provision of natural input for crop production (i.e.: crop provisioning service).

In line with the identification of the three pillars (economy, environment, society) of sustainability (UN, 1987), the sustainability scoreboard is composed of:

• the ecosystem service element (ES) which reports the ecological side, and specifically the ecosystem contribution (as percentage) in generating the raw crop;
• the market element (Mkt) which reports about the economic side, and specifically the role of the crop as intermediate and final consumption, and its importance in trading;
• the food supply element (FS) which reports about the societal side, and specifically the availability of food for domestic consumption in kilocalories per capita per day.

The crop provision accounts as reported in Vallecillo et al. (2019) and in La Notte et al. (2019) are assessed in physical and monetary terms. The monetary value attributed to ecosystem services is based on market prices, adopting the same rationale used for the resource rent approach. The advantage of such approach is the full consistency with the official monetary accounts. The drawback is that this valuation technique exposes crop provision estimates to the crop prices fluctuations that in turn affect ES values. To avoid that the costs structure and relative prices affect sustainability assessment, we use the ecosystem contribution coefficient based on the *emergy* approach, i.e. based on physical assessment.

Bear in mind that in the case of oats and potatoes, the Mkt indicator is calculated without the relative value of processed crops. Moreover, for sunflower and sugar beet no indicator was available for FS: the scoreboard in this case is only built for the Mkt and ES indicators.

The sustainability scoreboard of each crop is reported in detail in the following section. The scoreboard reports a heat map based on the ranking of the indicators on the left side, and a horizontal bar graph on the right side.

## 3 Results

**Fig. 2:**
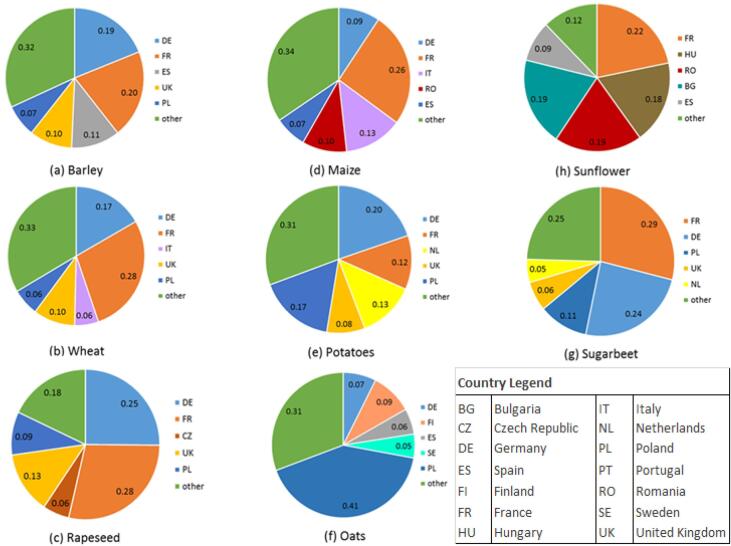
Production of selected raw crops in EU countries, year 2012 (source: ESTAT and FAOSTAT).
• on the other hand sustainability scoreboards report raking based on relative estimates in monetary terms (for Mkt and ES).

Each crop records different behaviours in the sustainability scoreboard and needs to be analysed separately. For each crop, the starting point are the agricultural statistics reported by Eurostat for the year 2012 (ESTAT, 2013). It is in fact important to read through different components of the same narrative:

• on the one hand agricultural statistics consider the total amount of production in tons (Fig. 2);

When these different set of information are jointly analysed, the overall picture becomes complex and multi-faced. Fig. 2 reports in percentage the production of selected raw crops in EU countries. France, Germany and Poland are among the top producers: in some cases, with important percentages (e.g. almost 1/3 of total production of potatoes in Germany, and of sugar beet, wheat and maize in France) in other cases, in line with other countries (e.g. rapeseed and oats). When interpreting these numbers which refer to the total production of each crop, the reader needs to keep in mind that we deal with values that are inevitably affected by the size of the country: Germany and France will always prevail on countries with a limited extension (even if extremely efficient in production). The reader also needs to keep in mind that we purely deal with “quantity”, no inference could be made on crop quality or management practices.

A different story can be outlined when looking at the sustainability scoreboards. Here for the market indicator (i.e. Mkt) the production is considered in relative values, so the size effect is avoided. Moreover, by using the GDP, the weight of the agricultural sector is reduced in those countries where secondary and tertiary sectors are highly developed.

**Fig. 3:**
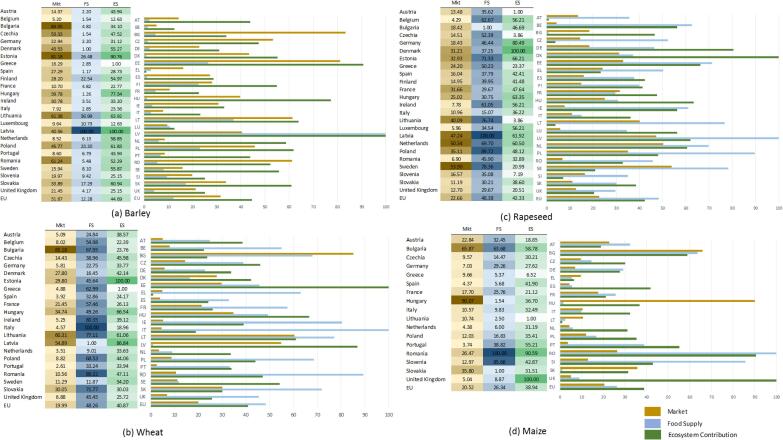
Sustainability scoreboard of barley, wheat, rapeseed and maize, year 2012.

Scoreboard results are analysed by crop and by country as follows. Fig. 3 shows the sustainability scoreboards for the crops where we are able to compute the three components of the Mkt indicator (i.e. raw crop, processed crop, trade), and the three indicators (i.e. Mkt, FS, ES) of the scoreboard: barley, wheat, rapeseed and maize.

As an example, let us consider barley and compare the number reported according to agricultural statistics (Fig. 2) and the sustainability scoreboard (Fig. 3). Germany and Poland are among the top five producers in Europe with respectively 19% and 7% of all production (Fig. 2). In the sustainability scoreboard Germany ranks low for the Mkt indicator (22.94), very low for the FS indicator (2.20) and low for the ES indicator (31.12); Poland ranks in the middle for the Mkt indicator (45.77) low for the FS indicator (23.20) and middle-high for the ES indicator (61.83) as shown in Fig. 3a. The difference between crop production reported in Fig. 2 and the Mkt indicator reported in Fig. 3 could depend on the importance that agriculture holds in the overall national economies, by the role of that crop within the agricultural sector.

Countries that record high scores in the sustainability scoreboard are Latvia (Mkt: 40.56, FS: 100, ES:100) and Estonia (Mkt: 81.18, FS: 26.48, ES: 90.76) as shown in Fig. 3a. The production of barley for these countries is aggregated together with the remaining 21 countries in EU and constitute the 32% of the total production (Fig. 2).

A high value of the ES indicator can depend on several factors: the use of extensive practices (e.g., less fertilizers and machinery), climatic conditions, field extension, etc.

The meaning of FS implies that domestic demand is fully covered by production. There could be many factors affecting the interpretation of this indicator within the scoreboard: e.g. from the traditional national diet, to the use of the raw crop as final consumption or as intermediate consumption (i.e. processed crop).

Although this analysis can be undertaken for each crop in each country, we here comment only few cases.

**Fig. 4:**
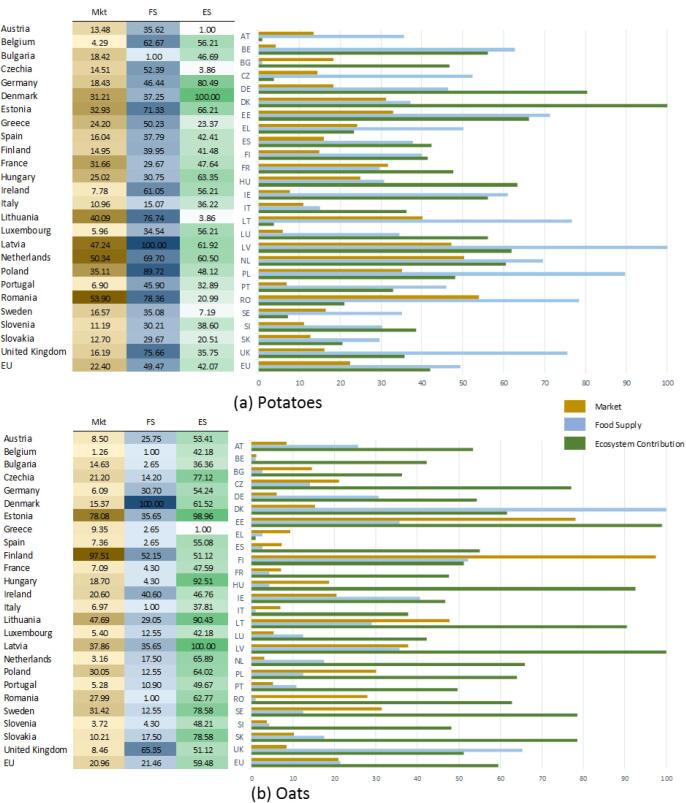
Sustainability scoreboard of potatoes and oats, year 2012.

Fig. 4 shows the sustainability scoreboards compiled for the crops where we are able to compute only two components of the Mkt indicator (i.e. raw crop and trade), and the three indicators (i.e. Mkt, FS, ES) of the scoreboard: oats and potatoes.

As an example, let us consider oats and compare the number reported according to agricultural statistics (Fig. 2) and the sustainability scoreboard (Fig. 4). Germany and Poland are again among the top five producers in Europe with respectively 7% and 41% of all production (Fig. 2). In the sustainability scoreboard Germany ranks very low for the Mkt indicator (6.09), middle-low for the FS indicator (30.70) and in the middle for the ES indicator (54.24); Poland ranks in middle-low for the Mkt indicator (30.50) low for the FS indicator (12.55) and middle-high for the ES indicator (64.02) as shown in Fig. 4b.

Countries that record high scores in the sustainability scoreboard are again Latvia (Mkt: 37.86, FS: 35.65, ES:100) and Estonia (Mkt: 78.08, FS: 35.65, ES: 98.96) as shown in Fig. 4b. The production of oats for these countries is aggregated together with the remaining 21 countries in EU and constitute the 31% of the total production (Fig. 2).

**Fig. 5:**
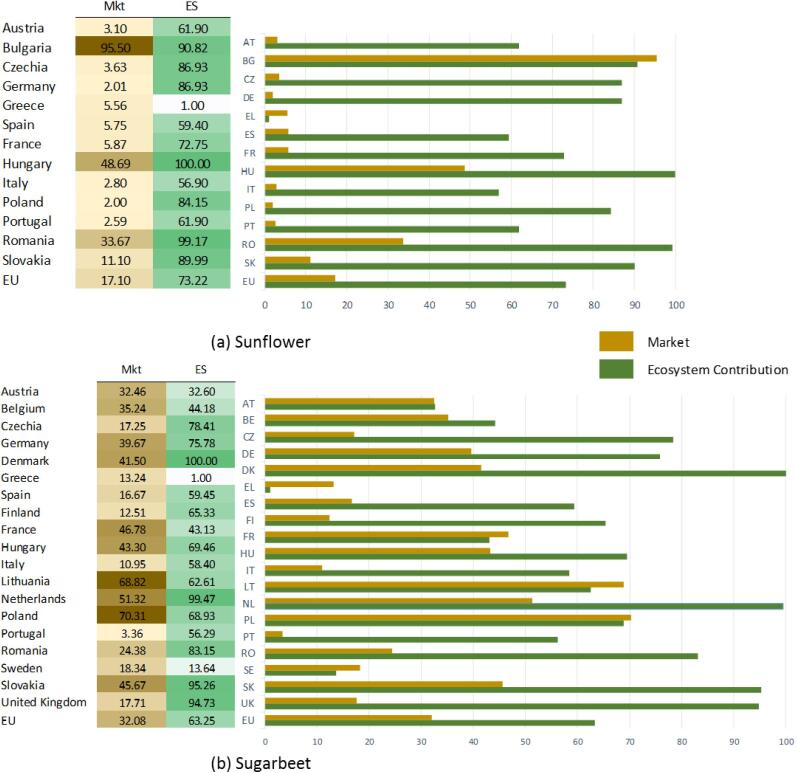
Sustainability scoreboard of sunflower and sugar beet, year 2012.

Fig. 5 shows the sustainability scoreboards compiled for the crops where we are able to compute three components of the Mkt indicator (i.e. raw crop, processed crop and trade), but only two indicators (i.e. Mkt and ES) of the scoreboard: sugar beet and sunflower.

As an example, let us consider sugar beet and compare the number reported according to agricultural statistics (Fig. 2) and the sustainability scoreboard (Fig. 5). Germany and Poland are again among the top five producers in Europe with respectively 24% and 11% of all production (Fig. 2). In the sustainability scoreboard Germany ranks middle-low for the Mkt indicator (39.07) and middle-high for the ES indicator (75.78); Poland ranks middle-high for both the Mkt indicator (70.31) and the ES indicator (68.93) as shown in Fig. 5b.

Differently from previous examples, countries that record high scores in the sustainability scoreboard are Denmark (Mkt: 41.50, ES:100) and Slovakia (Mkt: 45.67 and ES: 95.26) as shown in Fig. 5b. The production of sugar beet for these countries is aggregated together with the remaining 21 countries in EU and constitute the 25% of the total production (Fig. 2).

In summary, the sustainability scoreboard is able to provide a set of information which is complementary to the agricultural statistics that are commonly used. By considering relative values rather than total production in absolute terms, it is possible to estimate the importance of the crop (raw and processed, when available) with respect to the national economy as a whole: in the examples commented Germany and Poland are always among the top five producers according to conventional agricultural statistics (Fig. 2), but the role of the agriculture is more relevant in Poland than in Germany and this explains why the Mkt indicator is always higher for the former. By relating this market driven component with ecological and nutritional issues allows to picture an overall perspective: in the examples commented the highest ranking for FS and ES are always recorded for countries (such as Latvia, Estonia, Denmark and Slovakia) that are never reported as top five producers in agricultural statistics. This might imply that the current yield production is not currently aligned with sustainable agricultural practices within the food system.

## 4 Discussion

The importance of developing FAO-JRC combined tables lies in the consistent, separated reporting of ecosystem contribution and crop production without mixing, overlapping, and double counting. To use agricultural harvest as proxy for ecosystem service is not correct because the role of different agricultural practices could not be monitored through total yield; e.g.: intensive agriculture would generate higher biomass production than extensive practices, but at the same time would imply a lower ecosystem contribution. When looking at SDGs, it should be noticed that the sustainability scoreboard structure by including environmental (Natural Capital Input component), economic (agricultural production and related crop processed, imports and exports) and social variables (food availability) perfectly aligns with the three pillar dimensions for sustainable development, namely (i) the environment (ii) the economy (ii) the society as defined in the 2030 Agenda and its 17 SDGs as shown in Section 2, Methods, and in Fig. 1 – Structure of the FAO-JRC combined accounting table. Specifically, the sustainability scoreboard we developed may be useful in complementing existing monitoring approaches for several SDG indicators, including for instance those under Target SDG 2.4^14^ by tracking ecosystem contribution in crop provision and monitoring it over time together with the crop productivity and the food security.

Also, SDG 8, decent work and economic growth in its target 8.4, aims to decouple economic growth from environmental degradation which depends on over exploitation of resources and emission of pollutants. Therefore, by monitoring the combination (in terms of amount and degree) of ecosystem and human inputs in agricultural practices Authors aim to supply thought the JRC FAO crop provisioning accounting table and the sustainability scoreboard a crucial information framework, especially for rural developing countries.

**Fig. 6:**
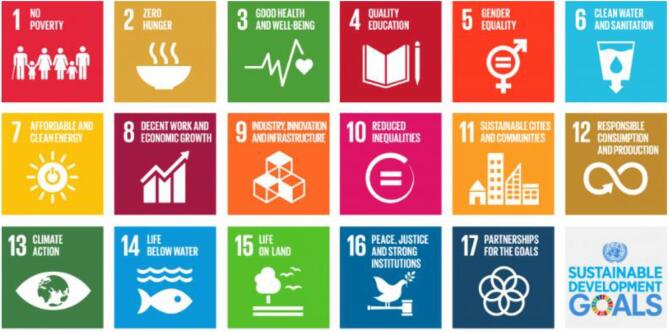
The 17 SDGs described in the 2030 Agenda for Sustainable Development (source: UN, 2020, at https://sustainabledevelopment.un.org/sdgs).

The accounting framework described in this paper also aim to contribute to SDG 12 and 15 (Fig. 6).

In fact, SDG 12, Ensure sustainable consumption and production patterns, directly refers to the sustainability of production practices and looks forward to consider the role of consumption (and thus consumers) in the crop-food supply chain. This perspective is fully covered in our analysis, which considers ecosystem contribution over raw and processed commodities.

Finally, FAO-JRC combined tables aim to contribute to SDG 15. Life on land, which promotes sustainable use of terrestrial ecosystems as they look backward to the state of terrestrial ecosystems that generate the natural resources needed by the economy and the society.

A sustainability scoreboard which aligns the three pillars together (ecosystem contribution, crop productivity and food security) could be an important tool. The sustainability scoreboard as well as the FAO-JRC accounting may in fact be used in a wider economic context to identify main food policy needs at national level. In fact, in addition to traditional agricultural statistics, it completes the picture by ranking the role of leading crops according to their economic importance for the country (in terms of consumption, transformation and trading), their ability to cover domestic demand, and the role of ecosystem contribution in the production process. Any agricultural policy that aims not only at economic growth but also at rural development could benefit from this stream of information. Moreover, the sustainability scoreboard may support national decision making because its component and its structure in line with the SNA, which is the key internationally agreed methodology to compute national economic indicators, as the GDP: the sustainability scoreboard enriches national economic perspective and analysis with environmental and social components and therefore facilitate a more accurate and sustainable policy decision making based on evidence.

From a methodological point of view, we first compute a composite indicator for the Market component (as it happens for very popular examples such as the Human Development Index,^15^ and the Environmental Performance Index^16^) and then we insert the composite indicator into a scoreboard (following a procedure adopted for example in the European innovation scoreboard^17^). The impossibility of using a unique index is justified by the likely trade-offs that might occur among the three pillars of sustainability. This article presents an attempt to present the complex information that underpins agricultural and food systems in a synthetic way.

The main limitation associated with this exercise was related to data availability. The primary need is in fact to fill data gaps: we had to make several assumptions to have some estimates and we could not build the scoreboard uniformly for all crops. At EU and UN level several initiatives are currently in place to improve data quality, for example by integrating national data collection with survey data (AGRIS initiative) or with geospatial information. There is the realistic hope that by time data availability accuracy and quality will improve, and this analysis extended to additional countries and commodities”. Nevertheless, the sustainability scoreboard as well as the JRC FAO accounting maybe used for macroeconomic context analysis to identify main food policy needs at national level. In this context the JRC mandate in terms of research and the FAO mandate of data producers to facilitate policy decision making perfectly fit. Another limitation of this study is that it does not include environmental and social cost of increasing agricultural production, which would require ad hoc analysis. This kind of assessment may raise the issue in terms of alarm to perform further analysis in the most critical cases, with the lowest ecosystem contribution and food supply scores.

## 5 Conclusion

Natural capital is a powerful instrument to measure natural resources and their contribution to national economy. For example, in this paper the role of ecosystems and their contribution by raw and processed crop to the GDP of European countries is analysed, well as the nutritional weight of the selected crops at country level. This type of analysis merges environmental, economic and social perspectives, facilitating analysis and policy decision making toward a sustainable food production.

As highlighted in the recent IPCC Special Report on climate change, the efficient use of natural resources for food production is even more urgent in the current climate change context, which affect all four dimensions of food security: food availability, food accessibility, food utilization and food systems. Natural resources are becoming increasing scares, while global food demand is increasing: there is therefore an urgent need for proper accounts of natural resources and their efficient use in food production.

The accounting framework as described above is in line with the SDGs Development Agenda, and in particular with Goal 2 End hunger, achieve food security and improved nutrition and promote sustainable agriculture, Goal 8 Promote sustained, inclusive and sustainable economic growth, Goal 12 Sustainable consumption and production pattern, and Goal 15 Promote sustainable use of terrestrial ecosystems. It may support traditional analysis on food supply chain by adding an ecological perspective, which is particularly important when considering emerging climate change call of efficient use of natural resources for food production.^18^

## Declaration of Competing Interest

The authors declare that they have no known competing financial interests or personal relationships that could have appeared to influence the work reported in this paper.

## Footnotes

1 FAO collects annual data on land use and agricultural practices (including organic agriculture) through the FAOSTAT land use questionnaire (http://www.fao.org/economic/ess/ess-home/questionnaires/en/). Information is disseminated with global coverage, over the time series 1961–2017 in FAOSTAT at http://www.fao.org/faostat/en/#data/RL.

2 SEEA EEA, Technical Recommendation, paragraph 5.3 Issues in the definition of ecosystem services.

3 Emergy (from “embodied energy”) of a product is defined as the total energy needed, directly and indirectly, to make that product. This approach allows from one side to measure natural and human inputs in common units of energy, and from the other side to derive ecosystem contribution to the agricultural output. The percentage of the yield that is directly attributable to the ecosystem contribution (*EcoCon*) can be derived in fact according to according to equation:*EcoCon*$=\frac{Natural\;Inputs}{\left(Natural\;Inputs\right)+\left(Human\;Inputs\right)}$, as described in, p. 15. Additional references to energy flows and agricultural biomass in provisioning services maybe found in Perez et Al, 2015.

4 Relevant crops may be different in different countries. Therefore, the crop list proposed in this accounting table is not mandatory.

5 CPC stands for Central Product Classification and constitutes a complete product classification covering all goods and services. It serves as an international standard for assembling and tabulating all kinds of data requiring product detail, including statistics on industrial production, domestic and foreign commodity trade, international trade in services, balance of payments, consumption and price statistics and other data used within the national accounts. It provides a framework for international comparison and promotes harmonization of various types of statistics related to goods and services. It is available on line at: https://unstats.un.org/unsd/classifications/unsdclassifications/cpcv21.pdf.

6 Main Databases used in this analysis are FAOSTAT (http://www.fao.org/faostat/en/#data) and Eurostat https://ec.europa.eu/eurostat/data/database, calibrated with JRC Common Agricultural Policy Regionalised Impact (CAPRI) model. Despite good data coverage from the above mentioned Databases, several initiatives are currently ongoing (including FAO International System for Agricultural Science and Technology, AGRIS and the FAO Geospatial information for sustainable food systems) that could contribute over time to data accuracy for the proposed natural capital perspective.

7 Selected variables sources are the FAO Statistical Framework for Environmental Economic statistics for Agriculture, Forestry and Fisheries (SEEA AFF) and the JRC Integrated project for Natural Capital Accounting (INCA).

8 Transforming our world: the 2030 Agenda for Sustainable Development, preamble “the SDGs. are integrated and indivisible and balance the three dimensions of sustainable development: the economic, social and environmental”, UN, A/RES/70/1, 21 October 2015. It was adopted by all United Nations Member States in 2015 At its heart are the 17 Sustainable Development Goals (SDGs), which are an urgent call for action by all countries – developed and developing – in a global partnership.

9 UN, A/RES/70/1, 21 October 2015, p 3.

10 In these tables, the column “supply from ecosystem to agriculture” in tons represent the amount of natural input disentangled by the provision of raw crops. This amount flows from the cropland ecosystem to the agricultural sector. Please refer to https://data.jrc.ec.europa.eu/dataset/df1e53e2-8c66-40d4-ae0b-068df7ed9969 for metadata information and methodology. The column “from ecosystem and human input to agriculture” (in tons) represents the raw crop that are used as raw crops, the column “from agriculture to manufacturing” (in tons) represents the processed crop. In processing other products countries might need to import raw materials and this makes it difficult to track a supply chain for each processed crop (it would require an ad hoc study). Raw crops, processed crops, import, export are extracted from FAOSTAT, please refer to http://www.fao.org/faostat/en/#data/QC for metadata information and methodology. For the food availability columns, please refer to http://www.fao.org/faostat/en/#data/FBS. For the FAO methodology through which from raw products are estimated corresponding amount of processed crops and food availability please refer to Food balance sheet methodology (http://www.fao.org/3/X9892E/X9892E00.htm).

11 This is the case: of Italy for barley, sunflower, sugar beet, rapeseed; of the Netherlands for maize and oats; of Ireland for potatoes and rapeseed; of Poland for sunflower; of Portugal for sugar beet; of Greece for rapeseed; of UK for maize.

12 The data are originally available in millions. They are transformed in thousands for operational purposes.

14 “By 2030, ensure sustainable food production systems and implement resilient agricultural practices that increase productivity and production, that help maintain ecosystems, that strengthen capacity for adaptation to climate change, extreme weather, drought, flooding and other disasters and that progressively improve land and soil quality” UN, A/RES/70/1, 21 October 2015, p 15.

15 Ref. http://hdr.undp.org/en/content/human-development-index-hdi.

16 Ref. https://epi.envirocenter.yale.edu/.

17 Ref. https://ec.europa.eu/growth/industry/innovation/facts-figures/scoreboards_en.

18 See also the IPCC special report on Climate change and Land, available on line at: https://www.ipcc.ch/report/srccl/.

## Footnotes

13 The use of equal weight assumes that there is no dominance of one element compared to others.
